# Smaller Fleas: Viruses of Microorganisms

**DOI:** 10.6064/2012/734023

**Published:** 2012-06-26

**Authors:** Paul Hyman, Stephen T. Abedon

**Affiliations:** ^1^Department of Biology, Ashland University, 401 College Avenue, Ashland, OH 44805, USA; ^2^Department of Microbiology, The Ohio State University, 1680 University Dr., Mansfield, OH 44906, USA

## Abstract

Life forms can be roughly differentiated into those that are microscopic versus those that are not as well as those that are multicellular and those that, instead, are unicellular. Cellular organisms seem generally able to host viruses, and this propensity carries over to those that are both microscopic and less than truly multicellular. These viruses of microorganisms, or VoMs, in fact exist as the world's most abundant somewhat autonomous genetic entities and include the viruses of domain Bacteria (bacteriophages), the viruses of domain Archaea (archaeal viruses), the viruses of protists, the viruses of microscopic fungi such as yeasts (mycoviruses), and even the viruses of other viruses (satellite viruses). In this paper we provide an introduction to the concept of viruses of microorganisms, a.k.a., viruses of microbes. We provide broad discussion particularly of VoM diversity. VoM diversity currently spans, in total, at least three-dozen virus families. This is roughly ten families per category—bacterial, archaeal, fungal, and protist—with some virus families infecting more than one of these microorganism major taxa. Such estimations, however, will vary with further discovery and taxon assignment and also are dependent upon what forms of life one includes among microorganisms.

## 1. Introduction

“So, naturalists observe, a flea

Hath smaller fleas that on him prey;

And these have smaller still to bit 'em;

And so proceed ad infinitum.

Thus every poet, in his kind,

Is bit by him that comes behind.” (Jonathan Swift (1733))

Swift's trophic progression does not, of course, proceed *ad infinitum*, but instead terminates with the viruses along, to a lesser degree, with the molecular parasites of those viruses. While viruses commonly are perceived especially as human pathogens and perhaps also as important parasites of domesticated animals or plants, the vast majority are hosted not by animals and plants but instead by “lesser” species, that is, by microorganisms. Indeed, animals and plants carry with them both extensive and diverse microbiota, and those organisms, in turn, are affected by their own microbiota, among which are included what can be described as the viruses of microorganisms.

Microorganisms typically are unicellular, and if they are eukaryotes, then they possess relatively few nuclei and often only one. The viruses of microorganisms therefore can be viewed predominantly as “unicellular organism parasites” [1]. This means, by and large, that individual cells of these organisms serve directly as targets for acquisition by freely diffusing environmental viruses. The inclusion of various colonial forms among microorganism—such as molds, colonial algae, and even bacterial arrangements and microcolonies—however complicates the idea of just what is and is not a microorganism. The dividing line between viruses of microorganisms and viruses of macroorganisms, that is, the distinction between what can be described as “VoMis” and “VoMas”, therefore is not absolute. Instead it is found somewhere on a spectrum between viruses that infect individual cells that live unassociated with clones of themselves, on one end, and viruses of true multicellular organisms on the other. Just what are viruses of microorganisms, using “VoM” as our preferred acronym, therefore is dependent on exactly how one defines microorganism.

In this review we take a somewhat inclusive approach towards defining “microorganism” and do so to consider VoMs that are associated with different types of hosts, which altogether span approximately three dozen named virus families (Table 1). These microorganism VoM hosts include all of domains Bacteria and Archaea. Within domain Eukarya, VoM hosts also include the microscopic members of the now-obsolete kingdom *Protista* along with the microscopic as well as pathogenic members of kingdom *Fungi*. These efforts to consider VoMs as a category of viruses—one that is somewhat distinct from the viruses of plants and animals both ecologically and in terms of general research priorities—we do particularly in light the recent formation of the International Society for Viruses of Microorganisms (ISVM.org), of which we both are founding executive members.

## 2. Introduction to Viruses

Human viral diseases have been known for millennia but tobacco mosaic virus was the first infectious agent to be identified as “ultrafilterable” (by Dmitri Iwanowski in 1892) and as a “contagium vivum fluidum” (by Martinus Beijerinck in 1898), that is, as what today we would describe as viruses. This was then followed by similar identification, by Friedrich Loeffler, of the foot-and-mouth disease etiology also as a virus, that is, as the first animal virus. While there are a few earlier papers that hint at the existence of bacterial viruses [2], more commonly known as bacteriophages or phages, the publications by Twort [3, 4] and d'Hérelle [5–8] are generally accepted as providing the first identification of these viruses, and also of VoMs. Definitive identification of viruses of microscopic fungi (mycoviruses), by contrast, did not occur until 1967, as reported by Ellis and Kleinschmidt [9], although an earlier mycovirus was demonstrated in macroscopic fleshy fungi, that is, mushrooms [10]. The first viruses of domain Archaea were identified in the 1970s [11], at approximately the same time the Archaea were becoming recognized as differing from Bacteria, or archaebacteria versus eubacteria as they were then distinguished. Finally, the first protist viruses were identified in the 1970s with amoeba viruses demonstrated in 1972 [12] and the first algal virus grown in culture in 1979 [13]. As with other groups of viruses, these protist virus identifications were preceded by a number of observations indicative of virus activity or the appearance of virus-like particles in cells [14, 15]. Ongoing discovery of viruses for both microbial and non-microbial organisms has led to our current understanding and confirmation that generally there are viruses for all forms of cellular life. While each virus is relatively specific in terms of host [16], the overall diversity of viruses and their number are vast, easily matching or exceeding that of their hosts.

Viruses are classified in terms of the type of hosts they infect, structural features associated with their virions, their genome type as well as specific gene sequences, and various details associated with the infection process. Virions tend to be quite consistent within a viral type and, by definition, consist of encapsidated nucleic acid. For the vast majority of viruses, that encapsidation involves one or more types of proteins, called capsomeres, forming into what are known as capsids. Furthermore, a large fraction of viruses possess lipids as part of the structure enclosing their nucleic acid genomes. Though in some cases these lipids are arrayed in what for animal viruses would represent atypical structures, more familiarly they are found as part of virus envelopes, which are lipid bilayers obtained from host membranes. Traditionally these different virion types have been distinguished into naked versus enveloped, though lipid-lacking versus lipid-containing can provide a broader distinction.

Virions also can be distinguished in terms of their size as well as shapes of their capsids, which for enveloped viruses are as found beneath the lipid envelope. For enveloped viruses, the overall shape of the virion is relevant as well. Standard shapes include spherical (as often seen with enveloped viruses), icosahedral (as often seen with naked viruses), filamentous, and tailed. Only a few VoM virions, by contrast, are pleomorphic. The tailed viruses are seen especially among bacteriophages, though certain archaeal viruses also have tails.

Viruses can possess genomes that consist of ssDNA, ssRNA, and dsRNA as well as the more familiar dsDNA. While most viruses possess genomes that are monopartite, that is, viral chromosomes consisting of only a single segment, multisegmented VoM genomes also exist. These include, for example, the tripartite, dsRNA genomes of the *Pseudomonas syringae* phage known as *φ*6 as well as a number of multipartite genomes among mycovirus and RNA viruses of protists. Genomes also can be differentiated in terms of size as well as into those that are linear, circular, or circularly permuted; the last is linear within the virion but nevertheless displaying linkage patterns as though they were circular. 

After virion binding to receptors, viruses insert their genomes into cells (uptake) and enter into a metabolically active state (biosynthesis) that represents the infection-proper of a cell. For most viruses, the entire cell is used to produce viral proteins and genomes and to assemble new virions. For the rest, especially viruses of eukaryotic cells, viral proteins may localize instead to the nucleus or a region in the cytoplasm to form areas of assembly sometimes called virion factories. Not all viral infections result immediately in virion assembly, and depending on the type of virus, at least one of three basic life cycle options exist: (1) lytic, (2) latent, or (3) chronic. These are (1) production and then release of virion particles in combination with destruction of the host cell to effect that release, (2) nonproductive infections in which viral genomes replicate along with their host cells (for phages this is known as a lysogenic cycle) and (3) productive infections with virion release that occurs without host cell destruction.

Many viruses are obligately lytic. Once infecting they coopt some or all of a cell's metabolic activity, replicate their genomes, produce capsid proteins, assemble new virions (maturation), and then lyse the cell to effect virion release, all without first adopting a latent or lysogenic state. These viruses invariably carry out these steps in the course of a successful infection, and, with bacteriophages as well as archaeal viruses, the term *virulent* is often used to describe them. We can also consider viruses that could be described as obligately chronic or, more generally, obligately productive. Successful infection by such chronically infecting viruses invariably results in production and release of virus progeny but, unlike the case with obligately lytic viruses, this release does not necessarily result, at least in the near term, in host destruction. 

Unlike obligately productive life cycles, many viruses instead can choose upon acquisition of a host cell between productive and latent infections. Such viruses include the temperate bacteriophages, which can display either lysogenic or productive infections upon infection and can also display productive infections after establishing lysogenic infections, following a process known as induction. During the latent state, the viral genome may be integrated into the host cell genome as a provirus or prophage but alternatively may exist as a plasmid-like episome. Whether as an integrated or episomal provirus, gene expression from the provirus genome often is limited to proteins needed to maintain the quiescent state as well as proteins used to monitor the host cell's metabolism.

## 3. Bacteriophages

The viruses of domain Bacteria, usually described as bacteriophages or phages, appear to be the most prevalent of VoMs, the most prevalent of viruses, and if we are willing to describe them as organisms, *perhaps* even the most prevalent of organisms. Total bacteriophage numbers on Earth—at least total VoM and virus numbers—may exceed 10^30^ virions, with potentially, for every cellular organism [20], more than one virus present. That estimation of 10^30^ virions also translates to an average of about 10^6^ viruses for every milliliter of sea water [21], which is perhaps an overestimation [22], though even greater numbers are present per gram of soil and at the surfaces of sediments [22, 23]. An assumption of 10^30^ total virions, most of them phages [22], therefore appears to be a reasonable baseline estimation of phage prevalence, with many authors suggesting 10^31^ or more.

What do such numbers mean? One way of viewing this prevalence is that it (perhaps) translates into as many as 10^24^ new phage infections occurring per second worldwide [24]. In addition, total phage mass could be in the range of 10^9^ metric tons (or more), assuming an average virion mass of 10^8^ daltons. The mass of 10^30^ phages thus could be roughly equal to the mass of one million blue whales [1], though as noted estimations can range even higher. Further, it has been estimated that all the world's phages lain end to end would form a chain that is roughly 10^6^ light years long [25, 26], which in turn is ten times the diameter of the Milky Way (about 10^5^ light years). Phages, if indeed these estimations are correct, thus likely play key roles in maintaining the diversity of bacterial communities, and perhaps particularly the diversity of cyanobacteria in marine environments [27], plus may impact climate in various ways [28]. In addition, all of the variations on infections as discussed in the previous section are seen among phages.

**Figure 1:**
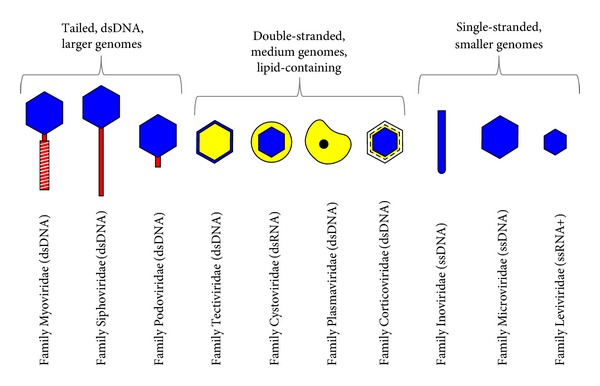
Bacteriophage families, morphologies, genome types, and relative genome sizes (keeping in mind that in many cases substantial variance is seen within categories, particularly for the tailed phages, in terms of both genome size and virion morphology). Phages are arranged in order of decreasing genome sizes. Blue coloration indicates capsids, red indicates tails, and yellow refers to lipids. Tailed phages are members of virus order Caudovirales. The figure is partially based upon those used in Ackermann [17], Hyman and Abedon [18], and Abedon [19]. Note that virion particles are not drawn to scale.

Phages can be classified to a first approximation in terms of their genome type and virion morphology [29], with genome size representing an additional interesting means of distinguishing among phages [19, 30]. At the higher end of genome size are the dsDNA tailed phages, members of virus order Caudovirales, which are thought to constitute the vast majority of phage types as well as individual virions. At the lower end are the single-stranded phages, whose genomes range in size instead from approximately 3.5 kb to 10.5 kb. The ssRNA phages (family Leviviridae), which are the smallest phages of all, are found at the lower end of this range. The single-stranded phages also include the members of family Microviridae, which have ssDNA genomes that are slightly larger than the ssRNA genomes of the leviviruses. In addition are the filamentous members of family Inoviridae, which are the larger of the ssDNA phages. In the middle, between single-stranded and tailed phages, are those that are double stranded, lack tails, and, interestingly, have virions that possess lipids. These ~10 kb to ~16 kb viruses consist of three dsDNA phage families (Corticoviridae, Plasmaviridae, and Tectiviridae) along with one double-stranded RNA phage family (Cystoviridae). See Figure 1 for illustration and summary of the major virion structural diversity seen among phages.

**Figure 2:**
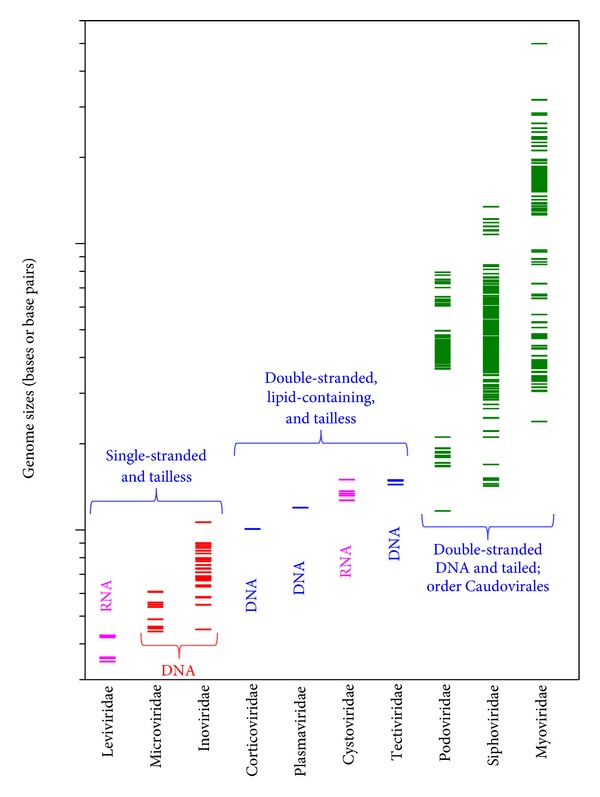
Comparison of phage genome sizes as differentiated by family. Genome sizes are as provided by NCBI (follow the Viruses link from http://www.ncbi.nlm.nih.gov/genome). Phage morphologies are provided also by NCBI but we defer to the International Committee for Virus Taxonomy given conflict between the two (http://www.ictvonline.org/). In addition, there are older sequences along with one newer sequence (phage G) that are not yet found on the above NCBI database page that we have included. These are for Enterobacteria phage SP (microvirus), Enterobacteria phage Fr (microvirus), Enterobacteria phage GA (microvirus), *Bacillus* phage G (myovirus), *Bacillus* phage PZA (podovirus), and *Streptococcus* phage SMP (siphovirus). Not included are genome sizes associated with unclassified phages. Total numbers of genomes included are as follows (if there are two numbers then the first is as found in the earlier version of this figure [19] and the second as found here): Leviviridae (10), Microviridae (17), Inoviridae (28→31), Corticoviridae (1), Plasmaviridae (1), Cystoviridae (5), Tectiviridae (4), Podoviridae (92→108), Siphoviridae (253→291), and Myoviridae (115→147). Purple refers to RNA genomes, red to ssDNA genomes, blue to dsDNA genomes as found in lipid-containing and tailless virions, and green, as indicated in the figure, are dsDNA in tailed and lipid-less virus particles.

Excluding satellite phages, as discussed in Section 7, the tailed phage genomes range in size from ~14 kb to ~500 kb, plus one oddly sized tailed phage, *Mycoplasma* phage P1, which possesses a genome size of less than 12 kb. Among the three phage members of virus order Caudovirales, and excluding *Mycoplasma* phage P1, the genome sizes range from ~16.5 to ~80 kb for members of phage family Podoviridae, ~14 kb to ~135 kb for phage family Siphoviridae, and ~24 kb to ~316 kb for phage family Myoviridae. In addition there is an outlier, also found in family Myoviridae, which is *Bacillus* phage G. Phage G has a genome size of approximately 500 kb. It is possible to compare these ranges graphically, as we do in Figure 2. Of interest, not only is there relatively little overlap in the genome-size ranges seen among various phage families but at this point in time distinctive gaps existing in terms of the genome sizes particularly within individual families of tailed phages.

The capsid for tailed phages, commonly described as the head, stores and protects the phage's genome so long as the phage is in its virion state and typically has an icosahedral form. The tail, by contrast, displays receptor-binding proteins and additionally holds proteins that facilitate genome entry into the bacterium and tailed phages fall into three classes (Figure 1). These include those that extend only a short distance from a site on one vertex of the capsid (family Podoviridae), those that are much longer but noncontractile structures (family Siphoviridae), and those that are both long and contractile (family Myoviridae). Among the nontailed virions, a few are filamentous (family Inoviridae), spherical (family Cystoviridae), or pleomorphic (family Plasmaviridae), while the rest are icosahedral, in many cases resembling tailed phage heads but without any tail. With filamentous phages, the receptor-binding proteins and genome entry effectors are located at one end of the filament while on icosahedral phages analogous proteins are found on the vertices of the capsid.

For additional consideration of bacteriophage biology, numerous monographs are available that review both basic and applied aspects of these organisms [31–36]. We also have published articles reviewing phage basic biology [18], ecology [37], evolution [30], and host range [16]. Phages are also noteworthy as biocontrol agents—phage therapy—that can be employed, for example, to combat bacterial infections in humans [38] (see in addition Section 8).

## 4. Archaeal Viruses

Archaeal viruses are at least as structurally diverse as bacteriophages, consisting of the same number of formally recognized virus families, ten [39], as there are formally recognized families for bacteriophages (Figures 1 and 2). This is extraordinary given that only about 45 archaeal viruses have been characterized [40] versus 100-*fold* greater numbers of bacteriophage isolates that have been isolated and then analyzed by electron microscopy [41]. The seemingly high diversity seen among archaeal viruses may reflect the many extreme environmental niches within which their hosts are found. Protein capsids adapted to high-salt environments for viruses infecting halophiles, for instance, may not be generally functional at the otherwise protein-denaturing temperatures at which various hyperthermophilic Archaea prefer to grow. Indeed, it is the latter especially that seems to have spawned the amazing variety of novel viral morphotypes that characterize the archaeal viruses. By contrast, the nucleic acid diversity of archaeal viruses is lower than that of bacteriophages as well as most other virus groups, consisting almost entirely of dsDNA with no RNA archaeal viruses currently known. 

Viruses of members of the archaeal taxon Euryarchaeota—which include methanogens, halophiles, and some of the thermophiles—appear to be largely members of virus order Caudovirales, that is, as is also the case for phages. These are the tailed viruses, which among archaeal viruses include members of families Siphoviridae and Myoviridae. Additional morphologies include icosahedral, “lemon-”shaped, and pleomorphic [40]. All have dsDNA genomes except for one, *Halorubrum* pleomorphic virus 1 (HRPV1), which has instead a circular ssDNA genome consisting of 7,048 nt. Among the dsDNA viruses of Euryarchaeota, genome sizes range from 8,082 nt for *Haloarcula hispanica* pleomorphic virus 1 (HHPV-1) to 77,670 nt for *Halorubrum* phage HF2.

**Figure 3:**
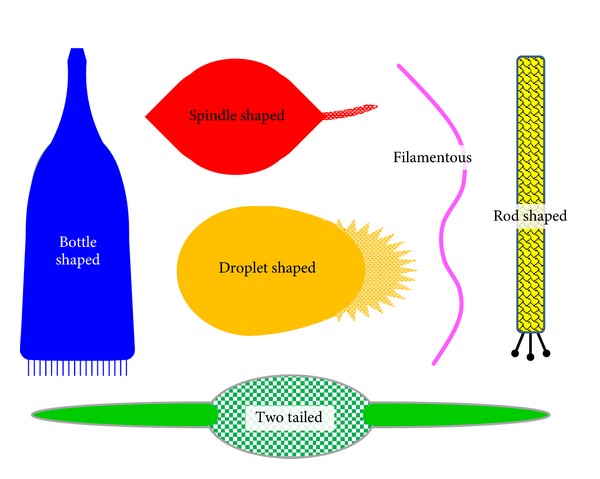
Schematic representation of various Crenarchaeotavirion morphologies. Reading clockwise from the left, bottle shaped (blue) is the morphology of family Ampullaviridae. The spindle shape is associated with family Fuselloviridae as well as the otherwise unassigned genus *Salterprovirus* (red). The enveloped Lipothrixviridae are filamentous (fuchsia), with various end-cap adornments not shown. There are four genera associated with this family, *Alphalipothrixvirus*, *Betalipothrixvirus*, *Gammalipothrixvirus*, and *Deltalipothrixvirus*, with the *Alphalipothrixvirus* virions somewhat broader relative to length than the others. The rod-shaped viruses, yellow with black ornamentation, are members of family Rudiviridae. Note the terminal fibers located in the figure at the bottom of the virion. The two-tailed virus (green) is classified as a member of family Bicaudaviridae. Its tails form morphologically only following virion release from its parental cell. Lastly, family Guttaviridae (orange, middle) possess droplet-shaped virions, shown with representations of fibers starburst shape found at their “tail” (right) end. Virions are not drawn to scale.

Viruses of the archaeal taxon Crenarchaeota, which includes hyperthermophiles as well as numerous additional species that grow instead in less extreme environments, possess a number of “unusual” virion morphologies. These include virions that are lipid containing which, by contrast, is a relative rarity among phages (Figures 1 and 2). Virion morphologies include bacilliform, bottle shaped, droplet shaped, filamentous, icosahedral, rod shaped, spherical, spindle shaped (also described as lemon shaped), and two tailed (Figure 3). All have dsDNA genomes that range in size from 5,278 nt for *Aeropyrum pernix* bacilliform virus 1 (APBV1) to 75,294 nt for *Sulfolobus *spindle-shaped virus 1 (STSV1), which is remarkably similar to the range seen among the Euryarchaeota viruses. See Table 2 for consideration of the taxonomy of this archaeal virus structural diversity.

In comparison with phages, there appear to be a greater fraction of isolates among archaeal viruses that have medium-sized genomes, that is, in the range of approximately 10 kb to 16 kb. Larger genomes nevertheless predominate, though much less so than they do among phages. In addition, and unlike phages, very large genomes, for example, >100 kb, are at best somewhat rare. The trend among both phages and archaeal viruses thus appears to be numerical domination by larger rather than smaller genome sizes, but with genome sizes for quite a number of phages (Figure 2) much larger than what so far has been seen with archaeal viruses.

In terms of life cycles, archaeal viruses are known which exhibit lytic infections as well as infections where virions instead “exits the host without causing cell lysis” [42, page 3687]. The latter, chronic infections a.k.a. “carrier state” when employed to describe archaeal viruses, is quite common among archaeal viruses infecting halophilic hosts [39]. Lytic infections are seen, not surprisingly, among members of order Caudovirales, that is, just as is the case with phage members of this order. Lytic infections otherwise do not appear to dominate among known archaeal viruses as they do among bacteriophages. In addition and also as with phages, there are latent infections (lysogens) involving either integrated proviruses or episomal proviruses. For recent reviews of archaeal virus biology, see [11, 39, 40, 43]. 

Interestingly, the Archaea appear to be lacking among known pathogens, although Archaea can be mutualistic symbionts. This striking absence has been blamed on the general lack of overlap between phage and archaeal virus host ranges [44] and compares, again strikingly, with the plethora of virulence factor genes which are known to be associated with numerous bacteriophages [45, 46]. As with bacteriophages, horizontal gene transfer nonetheless is prevalent *among* archaeal viruses, at least within individual virus taxa. From structural biology, as well as genomics (for the tailed archaeal viruses versus phages), some similarities have also been noted between archaeal viruses and eukaryotic as well as bacterial viruses [40, 43]. This similarity is perhaps indicative of the vestiges of a distant shared ancestry [39].

## 5. Viruses of Protists

Protists are broadly divided into the photosynthetic (algae) and nonphotosynthetic (a.k.a., heterotrophic, apochlorotic, or protozoans), though with a few taxa displaying both properties (i.e., mixotrophs, as seen among the euglenoids). All of the protist viruses that have been identified infect aquatic species, which in turn represent the majority of protists. Of both topical and scientific interest, a number of the protist viruses are among the largest viruses known. After discussing the viruses of the two groups of protists, we will conclude this section with a discussion of the evolutionary implications of large protist viruses. For recent reviews of protist virus diversity, see [47–51]. See Table 3 for a list of notable viruses of microbial protist species.

### 5.1. Early Evidence for Existence of Viruses of Protists

The identification of protist viruses was preceded by a number of observations suggesting their existence. For example, viruses had been suggested as the causative agents for algae population collapses as early as 1958 [14] but the first cultivation of an algal virus was not reported until 1979. Mayer and Taylor [13] were able to cultivate a virus on the marine phytoflagellate (nanoflagellate) *Micromonas pusilla.* We identify this as the first report though with two caveats. First, virus-like particles had been observed in algae cells and filaments as early as 1958 but not cultured, and second, a number of viruses infecting cyanobacteria, which were at the time considered algae, had been reported as early as 1963 [14]. Similarly, the first report of a virus cultured on a nonphotosynthetic protist was preceded by a number of observations of virus-like particles in electron micrographs [15]. These virus-like particles were seen in *Entamoeba, Plasmodia, Leishmania, *and other protozoans. Generally the observations were made in ultrastructural studies so no attempt was made to cultivate the virus even if live samples were still available. The first demonstration of passage of a virus of a nonphotosynthetic protist was of two lytic viruses infecting *Entamoeba histolytica* [12]. These viruses were found in an erratically growing culture, presumably via spontaneous induction of latent viruses, although viral particles were not always visible. Filamentous particles in the nucleus and icosahedral particles in the cytoplasm, however, were seen in some cells.

### 5.2. Viruses of Microscopic Algae

Difficulty in culturing has hampered the study of viruses infecting single-celled algae. As a consequence, while there have been a variety of virus-like particles observed within algae and other protists, only a small number have been characterized. Nevertheless, though fewer species of algal viruses have been characterized than bacteriophages, the algal virus diversity appears to be equally broad [50]. This likely reflects the fact that algal-virus hosts, the photosynthetic algae, are polyphyletic, ranging from diatoms and dinoflagellates to unicellular green algae. Also included are chlorella and related symbionts of paramecia, which also are protists, and hydras, which instead are animals [52]. Based on the detection of viruses and virus-like particles in aquatic environments via both microscopy or metagenomic sampling, there appear to be a large number of algal viruses, though few have been cultured. Among those few that have been cultured, the viruses of algae can be broadly divided into two groups—large and small. 

The archetype of the large group is the Mimivirus, which infects amoeba as we consider in the following section. Soon after the Mimivirus was isolated, other large, icosahedral dsDNA viruses infecting algae were identified. All of these algal viruses have double-stranded DNA genomes, with genome sizes ranging from 170–510 kb. These genomes may encode both proteins and nonprotein gene products such as tRNAs [52]. While virions are not as large as the amoeba giant viruses, they still tend to be larger than most bacteriophages, with icosahedral capsules having diameters of 110–220 nm, which is over twice the diameter of a typical tailed bacteriophage head capsid [47]. This large size immediately brings into question one of the standard definitions of a virus, that it be a filterable agent, as at least some of these viruses would be excluded by standard filtration [49]. It also likely explains why many surveys of waters for viruses did not identify the giant viruses previously, as filtration to remove bacteria and protists is a standard step in these procedures.

While morphologically similar, these viruses, grouped together in the family Phycodnaviridae, are quite diverse genetically, forming six or seven different genera [48, 53]. Analysis of a few core genes, though these genes make up less than 1% of the ORFs in these viruses, nevertheless indicates that these viruses, as a taxon, may be monophyletic. Further analysis links the giant viruses to viruses that infect non-photosynthetic protists (amoebae) as well as a variety of invertebrate and vertebrate animals such as the mammalian poxviruses. This group is often described as the nucleocytoplasmic large DNA viruses or NCLDV, but Raoult and colleagues have recently proposed that these viruses could be grouped into a single order, the Megavirales [49]. In addition to large size, these viruses have more complex virion structures, including internal membranes. Within this proposed Megavirales order, however, only the Phycodnaviridae family members infect various species of marine and freshwater alga as well as some algal symbiotes. 

Within the Phycodnaviridae, particular species employ all of the major viral life cycles, that is, lytic, latent, and chronic [48]. An example of a lytic phycodnavirus is PBCV-1, a chlorovirus—members of virus Phycodnaviridae genus *Chlorovirus*—that infects the chlorella symbiont of *Paramecium bursaria* (PBCV-1 in fact stands for *Paramecium bursaria* virus 1, as listed in Table 3). PBCV-1 is a smaller member of the group, with a 331 kb genome encoding approximately 400 proteins and 11 tRNAs [51]. A typical infection lasts 6–8 hours, ending in lysis that releases ~1000 progeny particles, although only about 30% of these can productively infect a new host. It is not clear if this lack of virion functionality is due to production of defective particles or if instead the efficiency of infection is low. In contrast, some of the phaeoviruses that infect filamentous brown algae (Phaeophyceae), viruses in this case of a macroalga, can produce a latent infection. More specifically, the EsV viruses that infect several *Ectocarpus* species, which are filamentous brown macroalgae, are able to latently infect the free-swimming gametes of the algae, integrating the viral genomes into algal chromosomes [54]. The provirus is then replicated as the algae grow. Virus induction and production of progeny typically occurs during spore and gamete formation, but in some cases induction may not occur, with vertical transmission of the provirus resulting instead (note that EsV stands for *Ectocarpus siliculosus* virus). Finally, the coccolithoviruses, which infect coccolithophores, that is, algae that are enclosed in calcium carbonate-scaled tests, are secreted continuously during infection. While not forming a long-lasting chronic infection, EhV-86 virions (*Emiliania huxleyi* Virus 86), for example, nevertheless shed 400–1000 virions over the course of infection [48, 53].

Small viruses of single-celled algae, by contrast, have approximately 4–25 kb single-stranded or double-stranded RNA or DNA genomes. Very few of these viruses have been either cultured or analyzed [47, 55]. Based on morphology, genome characteristics, and phylogenetic analysis, they nevertheless have been classified into multiple viral taxa that also contain animal viruses. These include order Picornavirales and family Reoviridae as well as some as yet unclassified viruses. In spite of the small numbers of species or perhaps as a result of a small number of widespread oceanic species, for several of these viruses, notably HcRNAV which infects the diatom *Heterocapsa circularisquama* (HcRNAVstands for* Heterocapsa circularisquama* RNA virus), multiple strains have been isolated from disparate locations. Less is known about the life cycles of many of the small algal viruses, but MpRV [56], RsRNAV [57] and HcRNAV [58] are all lytic (*Micromonas pusilla* reovirus, *Rhizosolenia setigera* RNA virus, and *Heterocapsa circularisquama* RNA virus, resp.). As a general rule, small RNA algal viruses tend to have a larger burst size than the large dsDNA viruses, with some burst sizes in the 10^4^–10^5^ per cell range [50], though for several of the small viruses even this information has not yet been determined.

### 5.3. Viruses of Protozoa

With the exception of amoebas, much less is known about viruses of the nonphotosynthetic (apochlorotic) protists, often less formally referred to as protozoa. In part, as with the viruses of algae, this likely reflects the difficulty in culturing protozoa. Of those protozoa viruses that have been identified, some are large dsDNA viruses related to the Phycodnaviridae, that is, the *Mimivirus* and related genera. Other protozoan viruses are mainly smaller RNA viruses including both single- and double-stranded RNA viruses. 

The first of these amoeba viruses to be identified was the Mimivirus. With a diameter of ~0.75 *μ*m, it was originally mistaken for a parasitic bacterium within the amoeba host [52]. The Mimivirus genome is about 1.2 Mb and has over 1000 genes, many encoding functions not seen in any other group of viruses. These include genes for macromolecular biosynthesis and proteins as previously only seen in cellular organisms with a role in translation [49]. Many of Mimivirus genes appear to have been acquired from eukaryotic, bacterial, or bacteriophage sources [59]. Other genes have no known homologues [49]. Subsequently, additional viruses in the Mimiviridae family and several more distantly related viruses have been identified (see Table 3). 

Garza and Suttle identified a virus capable of infecting two strains of a nanoflagellate in the *Bodo* genus [60]. It was only partially characterized but showed what is now recognized as morphology similar to Mimivirus and other related large viruses. Viral particles were visible in infected nanoflagellates two days after infections and cell death was observed between four and eight days after infection. In 2010, Fischer and colleagues genetically characterized this virus [61], which was renamed CroV (*Cafeteria roenbergensis* virus) because the host species is now recognized to be a zooplankton microflagellate, *Cafeteria roenbergensis,* rather than a *Bodo* species. The fully sequenced genome of ~730 kb puts it among the larger of these viruses. Phylogenetic analysis places CroV with the Mimiviridae. 

Two other large dsDNA viruses have been recently identified that form a separate family from the Mimiviridae. The Marseillevirus was found in a water sample from a cooling tower water tank in Paris and infects the same species of *Acanthamoeba* as the Mimivirus [62]. It has a 368 kb genome that includes, like members of the Mimiviridae, genes from eukaryotic, bacterial, and viral sources as well as giant virus core genes. There are sufficient differences in the core genes however, to indicate that the Marseillevirus is in a new family, designated the Marseilleviridae. Recently a second member of the family, the Lausannevirus, has been identified [63]. This virus has a 346 kb genome with 89% gene identity with the Marseillevirus.

All of the viruses of protozoa described above are large dsDNA viruses of amoeba. Takao and colleagues have isolated and characterized the first ssRNA virus infecting an apochlorotic protest [64]. This virus, designated SssRNAV, standing for *Schizochytrium* single-stranded RNA virus, infects several species of thraustochytrid, which are fungoid marine protists of the *Schizochytrium* genus. SssRNAV is a lytic virus with most cells killed within 36 hours after infection. The burst size has been estimated to be in the 10^3^–10^4^ range. The SssRNAV genome is in the same size range as those of the small RNA algal viruses, about 9,000 nt [65]. 

A number of dsRNA viruses have been identified mainly infecting pathogenic protozoa. The first of these was a virus infecting *Trichomonas vaginalis, *followed by viruses of *Giardia lamblia, Leishmania braziliensis, Eimeria *spp., and* Babesia *spp. [66]. These viruses all have linear, dsRNA genomes between 5 and 7 kb and icosahedral capsids. Perhaps reflecting the small genome size, the capsid is usually composed of multiple copies of a single capsomere protein. Over time, additional isolates of related viruses of these hosts have led to their being grouped together in the family Totiviridae which also includes several fungal viruses [67]. 

### 5.4. Evolution of the Large dsDNA Viruses of Algae and Protozoans

Discovery of the Mimivirus and other giant viruses led to speculation as to their evolutionary history and how their origin might fit with the origins of viruses in general. In addition to the papers cited below, additional discussion of the evolution of these viruses can be found in [52, 62, 68]. Three models for the origin of viruses have been proposed: (1) a degeneration model that hypothesized that the viruses evolved from cellular parasites that have become simplified over time; (2) the escape hypothesis that viruses grew from autonomous genetic elements and grew by acquisition of genes; (3) the primordial virus hypothesis that says that viruses evolved with cells at an early time in the history of life on Earth, and thus viruses can be said to represent a fourth domain of life [69]. This last model has also led to a debate in the literature as to whether viruses should be included in the tree of life at all; see Moreira and López-García [70] and follow-up responses. Various aspects of Mimivirus and related giant virus biology have been raised in support of all three of the viral origin hypotheses.

**Figure 4:**
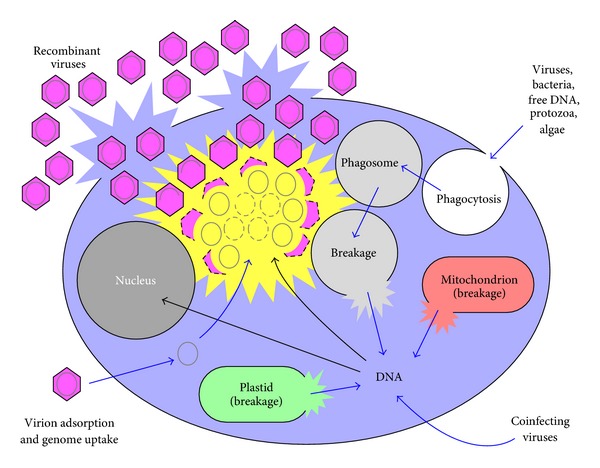
Summary of sources of DNA available for recombination by viruses of various protists. Any DNA that is able to find its way into a cell's cytoplasm, even accidentally, has a potential to become incorporated into preexisting DNA found within that cell, either as associated with the cell nucleus or with the virion factory structure (yellow) of infecting DNA viruses [62, 69, 71]. This illustration is also an elaboration on the “You are what you eat” hypothesis of Ford Doolittle [75] as elaborated further upon also by Andersson [76], Keeling and Palmer [77], and Abedon [30].

The large size and genetic complexity of the giant virus genome would seem to support the degeneration model, with giant viruses as a transitional form between cellular organisms and simpler viruses. In this view, the many genes in the giant viruses that do not correspond to any known genes of other organisms are presumably from the original ancestor parasite [71]. In contrast, several other groups have interpreted a core of genes that are exclusive to the giant viruses, that is, not acquired by horizontal gene transfer, as being indicative of viruses originating at an early time as cells were first evolving [53, 72]. Others see the giant viruses as growing from simpler viruses, of whatever origin, by horizontal gene transfer that has perhaps been aided by the environment of the amoeba interior where bacteria and single-celled protists are constantly being introduced and degraded in the course of amoeba feeding (Figure 4) [59, 68, 73]. 

As new and more distant members of the giant virus group are discovered, a consistent picture of a small core of unique genes with the remaining genes being a mixture of those acquired by horizontal gene transfer or of unknown origin (and often function) is emerging [74]. Most groups appear to be focusing on one of two models of viral origin corresponding to models (2) and (3) as listed above, the growth via horizontal gene transfer from simpler parasites or autonomous genetic elements (the “robber” hypothesis) model and the ancient origins (the fourth domain of life) model, respectively. The difficulty with distinguishing between these models is the reliance on a genome which by definition is exceedingly plastic, that is, subject to substantial gene exchange and evolution. For viruses, this difficulty is perhaps exacerbated by their ability to continue even following loss of what might otherwise be considered essential genes so long as they can substitute host functions. This suggests that a core of viral genes may be especially hard to interpret and it remains to be seen whether additional data can provide clarification. 

## 6. VoMs of Fungi

The viruses of fungi are known as mycoviruses and not all mycoviruses are necessarily of microorganisms. The fungus morphology—consisting of yeasts, pseudohyphae, hyphae, molds, mycelia, microscopic fruiting bodies, or macroscopic fruiting bodies, depending upon species, circumstances, and what portion of the organism one considers—in particular complicates such virus classification. So too, fungi can be free living or involved in symbioses that are mutualistic, commensalistic, or parasitic. We therefore begin this section with a brief discussion of what it means to be a microorganism before turning to consideration of mycoviruses generally and then as VoMs. Note that Göker et al. [78] provides an recent overview of the current state of mycovirology along with consideration of specific host-virus relationships. Earlier, authoritative reviews include that of Ghabrial and Suzuki [79], along with others as cited therein, and that of Pearson et al. [80]. There also are two earlier books on fungal viruses [81, 82]. 

### 6.1. Differentiating Fungal VoMis from VoMas

The definition of microorganism as applied to fungi can be particularly ambiguous. Indisputably yeasts are microorganisms as well as the pseudohyphae associated with certain dimorphic fungi such as *Candida* [113, 114]. Individual hyphae associated with fungi in general are also somewhat microscopic. That status of being quite small, however, changes as hyphae clump into collectively larger mycelia. Molds thus often are considered to be microorganisms whereas mycelia that give rise to macroscopic fruiting bodies, such as mushrooms, are not.

Size is not everything with regard to microorganism status since very small but well-differentiated organisms, such as rotifers, are typically not considered alongside protozoa, bacteria, and yeasts as microorganisms. Large colonies, including microbial mats made up of prokaryotes such as cyanobacteria that can form into stromatolites [115], on the other hand, are typically described as consisting of microorganisms, and this is even though the resulting structures can be clearly discrete as well as macroscopic, spanning up to meters in dimensions particularly in the fossil record [116]. Such hosts, in other words, collectively are “macro” but nevertheless consist of individual microorganism cells. As seen with the brown algae described above (Section 5), organisms that can grow into much larger as well as truly multicellular forms also can be infected while still existing as single cells. That is, these latter hosts while at some point “micro” nevertheless are not microorganisms.

Cellular differentiation along with morphological complexity thus can play roles in distinguishing microorganisms from “macroorganisms”, with the former usually small and always less morphologically complex and the latter usually not too small and always are morphologically more complex. Yeasts and molds therefore are considered to be microorganisms with little ambiguity while the fleshy fungi, a.k.a., macrofungi, are not. There also is a tradition in microbiology to consider pathogens to be microorganisms, and to a certain degree this holds for eukaryotic parasites such as pathogenic protozoa along with helminths as well. Of particular relevance, there are a substantial number of especially plant pathogenic fungi for which viruses have been identified.

Note as a caveat that Dawe and Kuhn [109] isolated a dsDNA virus infecting *Rhizidiomyces* sp. that is routinely reported as a mycovirus. *Rhizidiomyces* sp., however, is a water mold (oomycetes). Water molds, though superficially resembling fungi, including serving as plant pathogens, are now known to be protist descendants of algae [117]. Though water molds are traditionally grouped with true fungi in mycology—resulting in application of the term “mycovirus” to them in fact *not* being a misnomer—phylogenetically the viruses of water molds should be included among those reviewed in the previous section as protist viruses (Table 3). What then is known of the mycoviruses of yeasts, *true* molds, and pathogenic fungi?

### 6.2. VoMi of Fungi

Contrasting the diversity of viral types seen among other organisms, that is, the animal viruses, plant viruses, other nonmycovirus viruses of eukaryotes, bacteriophages, and particularly archaeal viruses (which are all DNA viruses), mycoviruses had been thought to consist solely of RNA viruses. The RNA genomes typically are double stranded, and some of those are multisegmented. Single-stranded RNA mycoviruses also exist, however, which are consistently plus stranded as well as monopartite (as one also sees among the ssRNA bacteriophages, i.e., with the Leviviridae). These RNA viruses furthermore can be differentiated into ten or more virus families: Alphaflexiviridae, Birnaviridae, Chrysoviridae, Gammaflexiviridae, Megabirnaviridae, Metaviridae, Partitiviridae, Pseudoviridae, Reoviridae, and Totiviridae. This list presumably will grow with time since recently, for example, Yu et al. [112] reported the isolation of a 2,166 base ssDNA geminivirus-like mycovirus, *Sclerotinia sclerotiorum* hypovirulence-associated DNA virus 1.

The morphology of mycovirus virions is predominantly isometric and less commonly spherical with double-shelled virions or instead filamentous. A mushroom virus also exists that is bacilliform in shape (mushroom bacilliform virus; family Birnaviridae). Mycovirus virions also are largely naked rather than enveloped. Unencapsidated infectious RNAs also exist, which are members of families Endoviridae and Narnaviridae. These are reminiscent at least superficially of the ssRNA viroids seen with plants [118]. Fungus-associated families, though, are roughly fifty-times larger in terms of genome size (>10,000 nt versus >200 nt), members of family Endoviridae are double stranded rather than single stranded, and both, unlike viroids, possess genes. Also of interest are members of family Hypoviridae, which are “encapsidated” within structural-protein-less pleomorphic vesicles [29].

Mycovirus infections are latent and, from the perspective of host phenotype, often inapparent, that is, symptomless or cryptic. In a number of cases, however, they modify the pathogenicity of disease-causing fungi either downward (hypovirulence) or upward (hypervirulence). The latter is perhaps similar in general effect to the ability of certain also latently infecting bacteriophages to increase the pathogenicity of their bacterial hosts [119]. Mycoviruses additionally can affect host phenotypes in more subtle ways. This is seen with mycovirus-associated “killer phenotypes” in some yeasts, which are reminiscent in their impact on yeast competitiveness [120, 121] to the impact of bacteriocins on bacteria [122]. Of additional interest are the following: detection of novel mycoviruses can often involve identification of presumptive viral RNA, and particularly it is dsRNA that is looked for in these assays; RNA silencing effected by certain host fungi may serve as an antimycoviral defense [123]; *Saccharomyces cerevisiae* viruses Ty1, Ty2, and Ty3 are also commonly described as both retroviruses and retrotransposons [124].

Mycoviruses are “transmitted intracellularly during cell division, sporogenesis, and cell fusion, but apparently lack an extracellular route for infection” [79, page 353]. They also are not known to be transmitted by vectors. For these reasons some authors prefer to describe mycoviruses as “viruslike” rather than strictly as viruses. This raises the interesting question of whether in fact viruses must have an extracellular phase, protein capsids, or indeed any encapsidation, as can be lacking among fungal “viruses”, in order to be strictly described as viruses.

Between NCBI complete genomes and the ICTV list of virus names and taxa, there are over 90 mycoviruses. The majority of these viruses appear to infect plant pathogenic fungi [79, 80] while 15 infect yeasts and five saprophytic molds. Hosts to yeast viruses include *Saccharomyces cerevisiae*, *Schizosaccharomyces pombe*, and *Candida albicans*. Various reviews of yeast mycoviruses and their biology can be found elsewhere [124, 125]. Hosts to mold viruses include *Penicillium* spp. as well as *Aspergillus ochraceus*, which is infected by *Aspergillus ochraceous* virus. A summary of the diversity seen among especially NCBI and/or ICTV indexed mycoviruses is presented in Table 4.

In a recent study of plant-associated endophytic fungi and their viruses, Feldman et al. [126] found that about 10% of 225 fungal samples studied were associated with mycoviruses, which they grouped into 16 different taxa based on RNA-genome detection. Also recently, Ikeda et al. [127] have identified virus-like RNA associated with a mycorrhizal fungus. Indeed, though a PubMed search on mycovirus or mycoviruses yields only 150 hits (searched on May 28, 2012), 13 of those hits have 2012 dates and 17 have 2011 hits, suggesting a growing interest in these viruses. 

In light of the ability of mycoviruses to infect fungal pathogens, these viruses have been suggested as therapeutic agents against fungal pathogens of both humans [128] and plants [80]. Such approaches are complicated in no small part by the difficulty in effecting extracellular transmission with mycoviruses as well as the typical latent infection displayed by these viruses. The ability of many mycoviruses to reduce the virulence of their hosts (hypovirulence), however, provides a possible therapeutic or biocontrol goal [129].

## 7. Viruses of Other Viruses

Including viruses in the tree of life or even as living organisms is debatable [130]. With that caveat in mind, consider the existence of viruses that serve as obligate parasites of other viruses, that is, viruses that successfully produce virion progeny only within cells that have been infected by another virus. These viruses are typically described as satellite viruses but more recently the term virophage has been introduced for some [131]. Since the “host” virus may have reduced production of progeny when the satellite virus is present, the satellite virus can be considered a parasite on the host virus; that is, the typical virus gains while the host loses interaction [131, 132]. While there are many satellite viruses of plant and animal viruses, as of mid 2012 only six satellite viruses utilizing VoMs are in the literature, although metagenomic analysis of water samples suggests there may be many more [133]. In addition, there are defective interfering (DI) particles as well as DI particle-like viral mutants that similarly can serve as parasites particularly of virus infections and which have been identified as parasites of phages [134] as well as of mycoviruses [79]. One can make a distinction, however, between DI particles, which contain defective genomes of the same species of virus that is being parasitized, and satellite viruses/virophages which are clearly not the same species, if sometimes related, as the virus being parasitized. 

Bacteriophage satellite phage P4 was isolated in the 1960s as a temperate phage that could produce virion progeny only when infecting *E. coli *strains that are lysogenic for bacteriophage P2 or a few related phages [135]. P4 is smaller than P2 in both genome and capsid size, with a capsid containing a mix of P2 and P4 proteins. In the absence of P2 genes, P4 is still able to infect *E. coli,* but it can only form a latent prophage, which may be integrated or exist as a stably replicating, multicopy plasmid episome. Infection of a P4 lysogen with P2 leads to induction of the P4 prophage and subsequent P4 production and release.

A similar situation exists for satellite virus *φ*R73, which was also identified in some strains of *E. coli* and is also dependent on bacteriophage P2 for lytic growth. *φ*R73 contains genetic elements that are clearly derived from phage P4, the other satellite virus of P2, but also contains genes characteristic of a retrotransposon, including integrase and reverse transcriptase [136, 137]. Because of this combination of phage and retrotransposon elements, *φ*R73 is sometimes described as a retronphage. The transposon elements allow the 12.7 kb genome to integrate into a specific site in the *E. coli *genome, in the selenocystyl tRNA gene.

The case of the one known archaeal satellite virus also contains some ambiguity similar to that of *φ*R73. With pSSVx, the satellite virus also has plasmid-like properties that go beyond forming an episome and has some sequence identity with other plasmids found in *Sulfolobales*, an extreme thermophilic archaean in the Crenarchaeota taxon [138]. Either SSV1 or SSV2 Fuselloviridae—both spindle-shaped viruses with circular dsDNA genomes—can act as helper viruses to package the pSSVx genome. Even though the pSSVx genome is only about 1/3 the size of helper virus genome (5.7 kb versus 15.5 kb for SSV1), the virion particle size appears to be the same for both and it has proven difficult to separate the virions based on size. Also unlike the P4 case, where only P4 virions could be isolated without any associated P2 helper particles, the virions of pSSVx and helper virus were always released together.

Virophages is the term favored for satellite viruses of large DNA viruses of protists and some have argued that virophage should be a separate class from satellite viruses [139]. Several have been identified. The first seen was the Sputnik virus, an 18 kb, dsDNA virophage that infects amoeba infected with the Mamavirus, another member of the Mimiviridae [131]. Unusually, the virion particle also contains most of the viral RNAs, presumably to aid in takeover of the Mamavirus virion factory complex within the host amoeba [139]. Production of Mamavirus virions is reduced with Sputnik infection, indicating that it is a true parasite of the Mamavirus. More recently, the Mavirus virophage has been identified in association with CroV, the large DNA virus of *Cafeteria roenbergensis* [140]. This virophage has a slightly larger genome than Sputnik, with 19 kb of dsDNA. It also has genes that are related to several transposons, suggesting either a vertical evolutionary relationship or significant horizontal gene transfer.

Yau and colleagues recently published a metagenomic analysis of samples taken from an Antarctic lake over a two-year period that included a virophage [133]. Designated OLV (Organic Lake virophage), this virophage is likely associated with a phycodnavirus (which infect algae) rather than an amoeba virus, an inference based on the phycodnavirus genome sequences identified in the same samples. They were able to reconstruct the 26.4 kb genome and found that while it had some relationship to Sputnik, it clearly had undergone extensive horizontal gene transfer as well, containing genes related to many other species. Finally, they examined water samples from other lakes, including two tropical lakes and found sequences indicating the presence of other virophages in these environments as well.

## 8. VoM-Mediated Biocontrol

Mycoviruses have been proposed as a means of protecting plants from pathogenic fungi [79, 129], and a small literature exists on the potential for employing viruses to control algae or cyanobacterial blooms [141, 142]. By far the greatest exploitation of the idea of viruses as biocontrol agents, however, is seen with the biocontrol of heterotrophic bacteria. Depending on the context of this phage-mediated biocontrol, it is also commonly referred to a phage therapy, that is, particularly when used in a medical or veterinary context, though there exist agricultural- and food safety-associated “biocontrol” applications of phages as well [143]. For lists of reviews on the subject, see [144] and the web site, http://phage-therapy.org/. For recent discussion of the use of phage therapy to treat human disease, see Kutter et al. [145] and Abedon et al. [38]. Here we provide a brief overview of the technology.

Phage therapy was invented soon after the discovery of phages, with the first human trials carried out no later than 1921 [38]. This use was not surprising given d'Hérelle's initial belief that phages were an “immunity microbe” whose appearance marked the resolution of a bacterial infection [7]. At that time the utility of phages as antibacterial agents was difficult to ignore given their apparent safety in combination with a relative lack of antibacterial drugs, particularly since penicillin would not be discovered until 1928. Various factors would serve to reverse this trend, however, not least of which was a relative lack of understanding of phage biology along with the commercialization of antibiotics. As a consequence, the 1940s and beyond were not kind to the practice of phage therapy in much of the Western hemisphere [38]. Nonetheless, pockets of phage therapy enthusiasm persisted, most notably in the former Soviet Union as centered in the Soviet state of Georgia, in France, and in Poland. Since the mid 1990s, in response to a combination of the opening of Soviet Bloc countries and the rise in concerns over antibiotic resistance among bacteria, there has been a resurgence in interest in phage therapy both academically and among various Western start-up companies (for a current list of companies focusing on the therapeutic use of phages, see ISVM.org). Most notably, phages are currently sold commercially for biocontrol purposes by the Utah company OmniLytics, the Dutch company Micreos Food Safety (formerly EBI Food Safety), and the Maryland company Intralytix. Notable also is AmpliPhi (formerly Biocontrol) which has progressed furthest among phage therapy companies in terms of clinical trials, employing anti-*Pseudomonas* phages against chronic otitis.

The advantages of phages as antibacterial agents are numerous, as recently considered by Loc-Carrillo and Abedon [146] along with Curtright and Abedon [147]. Above all is their safety when compared with alternative infection-control agents, and this is particularly so given adequate phage characterization and purification prior to use [148]. The result is what can be a highly effective, relatively inexpensive, and easily obtained antibacterial agent that has been administered to thousands patients with few reported side effects, which is effective even against antibiotic-resistant bacteria and which is often effective even against chronic bacterial infections [38] and biofilms [149, 150]. In a world increasingly anxious about both the use of antibiotics and resulting antibiotic resistance and with numerous individuals who both suffer and die from antibiotic-resistant infections even given the best treatment modern chemotherapies can provide, it is of keen interest that not only are phages potentially available to augment antibiotic therapies but in fact have been routinely employed for just that, with documented success observed over the course of decades in the former Soviet Union and Poland [38, 145].

VoM-mediated biocontrol has been suggested and studied in the laboratory also for use against algae blooms, which can negatively impact aquatic environments when nutrient runoff triggers a massive overgrowth that results in what are termed toxic blooms, red tides, or harmful algal blooms. These can have a significant negative impact on other aquatic life, depleting water of nutrients as well as via toxin production by some algae such as dinoflagellates [151]. The viruses of algae therefore can have equally significant effects in their role as algae predators and there have been several observations supporting a role for viruses in the ending of some algal blooms [50]. For example, blooms caused by *Heterosigma akashiwo* Raphidophyceae algae are sometimes observed to breakdown suddenly. At the time of those breakdowns, the proportion of cells containing virus-like particles increases, as observed via electron microscopy [152]. Even without population depletion, viruses can affect populations of host algae. A large dsDNA virus, HaV, specific for *H. akashiwo* has been isolated from ocean regions where *H. akashiwo* is found. Tarutani and colleagues observed levels of *H. akashiwo *and HaV during the growth and breakdown of a bloom near the coast of Japan [152]. They found that the virus caused a shift in the population dynamics of the algae between virus-resistant and virus-sensitive strains. Likewise, levels of viruses of several species have been found to vary seasonally along with their host algae [50].

## 9. Conclusion

For the sake of uniformity in nomenclature, it recently has been suggested that viruses might be distinguished, at the highest level and in terms of their host organisms, into bacterioviruses, archeoviruses, and eukaryoviruses [39]. Operationally, however, viruses have long been differentiated into (1) those that infect animals as studied under the guise of biomedicine, (2) those that infect plants as studied under the guise of agriculture and plant pathology, and (3) essentially everything else. This everything else includes bacterioviruses, archeoviruses, and a diversity of eukaryoviruses, with currently three dozen or so virus families making up this “everything else” and which to a large extent are distinctly different from those viruses that infect animals and plants.

Though by no means as easily defined a virus category as are bacterioviruses, archeoviruses, and eukaryoviruses, these other viruses consist predominantly of the viruses of microorganisms. In addition to domains Bacteria and Archaea, these microorganisms include unicellular and non-coenocytic eukaryotes, most or all organisms that do not exhibit true multicellularity, and most or all organisms that can be described as pathogens. Not only are the viruses of these organisms more numerous than animal or plant viruses, but they likely also are more genetically diverse [153, 154]; see also [155]. In light of this relevance, the goals of this paper have been to provide an effort towards defining just what viruses of microorganisms are and then to consider the extent of especially their taxonomic diversity. Our general conclusion is that there is quite a bit more to the virosphere than *just* the viruses of animals and plants.

## 10. Note

Of especial interest to readers of this review, Ackermann and Prangishvili have published a survey bacteriophages and archael viruses based on electron microscopic imagery [156].

A recent presentation has informed us of a new virophage, designated Sputnik2, that was noted but not described by Cohen and colleagues [157]. It was found with a novel giant virus of the Mimivirus group in the contact lens solution of a patient who had an ocular amoebic infection. C. Desnues presented a description of this Sputnik-like virophage at the Viruses of Microbes meeting in Brussels, July, 2012.

## Figures and Tables

**Table 1:** Summary of current classification of viruses of microorganisms^1^.

Family^2^	Genera	Genome	Microbe^3^	Additional
Ampullaviridae	*Ampullavirus*	dsDNA	Archaea	Bottle shaped
Bicaudaviridae	*Bicaudavirus*	dsDNA	Archaea	Lemon shaped prior to growing two tails
Clavaviridae	*Clavavirus*	dsDNA	Archaea	Bacilliform
Corticoviridae	*Corticovirus*	dsDNA	Bacteria	Lipid containing
Fuselloviridae	*Fusellovirus*	dsDNA	Archaea	Spindle shaped
Globuloviridae	*Globulovirus*	dsDNA	Archaea	Spherical
Guttaviridae	*Guttavirus*	dsDNA	Archaea	Droplet shaped
Lipothrixviridae	*Alphalipothrixvirus*	dsDNA	Archaea	Filamentous
	*Betalipothrixvirus*	dsDNA	Archaea	Filamentous
	*Deltalipothrixvirus*	dsDNA	Archaea	Filamentous
	*Gammalipothrixvirus*	dsDNA	Archaea	Filamentous
Mimiviridae	*Mimivirus*	dsDNA	Protista	Complex, lipid-containing, icosahedral capsid
(C.) Myoviridae	[numerous genera]	dsDNA	Bacteria	Contractile tail
	Φ*H-like viruses*	dsDNA	Archaea	Contractile tail
Phycodnaviridae	*Chlorovirus*	dsDNA	Protista	Icosahedral
	*Coccolithovirus*	dsDNA	Protista	Icosahedral
	*Prasinovirus*	dsDNA	Protista	Icosahedral
	*Prymnesiovirus*	dsDNA	Protista	Icosahedral
	*Raphidovirus*	dsDNA	Protista	Icosahedral
Plasmaviridae	*Plasmavirus*	dsDNA	Bacteria	Lipid containing
(C.) Podoviridae	[numerous genera]	dsDNA	Bacteria	Short tail (non-cont.)
Rudiviridae	*Rudivirus*	dsDNA	Archaea	Rod shaped
(C.) Siphoviridae	[numerous genera]	dsDNA	Bacteria	Long tail (non-cont.)
(C.)	*ψM1-like viruses*	dsDNA	Archaea	Long tail (non-cont.)
Tectiviridae	*Tectivirus*	dsDNA	Bacteria	Lipid containing
[unassigned]	*Dinodnavirus*	dsDNA	Protista	Complex, lipid-containing, icosahedral capsid
[unassigned]	*Salterprovirus*	dsDNA	Archaea	Spindle shaped
Inoviridae	*Inovirus*	ssDNA	Bacteria	Filamentous
	*Plectrovirus*	ssDNA	Bacteria	Filamentous
Microviridae (G.)	*Bdellomicrovirus*	ssDNA	Bacteria	Icosahedral
	*Chlamydiamicrovirus*	ssDNA	Bacteria	Icosahedral
	*Spiromicrovirus*	ssDNA	Bacteria	Icosahedral
Microviridae	*Microvirus*	ssDNA	Bacteria	Icosahedral
[unassigned]	*Bacilladnavirus* ^ 4^	ssDNA	Protista	Icosahedral
[unassigned]		ssDNA	Archaea	Lipid containing
[unassigned]		ssDNA	Fungi	Spherical or icosahedral (geminivirus like)
Chrysoviridae	*Chrysovirus*	dsRNA	Fungi	Icosahedral
Cystoviridae	*Cystovirus*	dsRNA	Bacteria	Lipid containing
Endornaviridae	*Endornavirus*	dsRNA	Fungi	Unencapsidated
	*Endornavirus*	dsRNA	Protista	Unencapsidated
Hypoviridae	*Hypovirus*	dsRNA	Fungi	Pleomorphic cytoplasmic vesicles
Megabirnaviridae	*Megabirnavirus*	dsRNA	Fungi	Spherical
Partitiviridae	*Partitivirus*	dsRNA	Fungi	Icosahedral
	*Cryspovirus*	dsRNA	Protista	Icosahedral
Reoviridae (Se.)	*Mimoreovirus*	dsRNA	Protista	Icosahedral
Reoviridae (Sp.)	*Mycoreovirus*	dsRNA	Fungi	Spherical, double shelled
Totiviridae	*Giardiavirus*	dsRNA	Protista	Icosahedral
	*Leishmaniavirus*	dsRNA	Protista	Icosahedral
	*Totivirus*	dsRNA	Fungi	Icosahedral
	*Trichomonasvirus*	dsRNA	Protista	Icosahedral
	*Victorivirus*	dsRNA	Fungi	Icosahedral
[unassigned]	*Rhizidiovirus*	dsRNA	Protista	Icosahedral
(T.) Alphaflexiviridae	*Sclerodarnavirus*	ssRNA (+)	Fungi	Filamentous
	*Botrexvirus*	ssRNA (+)	Fungi	Filamentous
Alvernaviridae	*Dinornavirus*	ssRNA (+)	Protista	Icosahedral
(P.) Bacillariornaviridae^5^	*Bacillariornavirus* ^ 6^	ssRNA (+)	Protista	Icosahedral
(T.) Gammaflexiviridae	*Mycoflexivirus*	ssRNA (+)	Fungi	Filamentous
(P.) Labyrnaviridae^7^	*Labyrnavirus* ^ 8^	ssRNA (+)	Protista	Icosahedral
Leviviridae	*Allolevivirus*	ssRNA (+)	Bacteria	Icosahedral
	*Levivirus*	ssRNA (+)	Bacteria	Icosahedral
Marnaviridae	*Marnavirus*	ssRNA (+)	Protista	Icosahedral
Pseudoviridae	*Hemivirus*	ssRNA (+)	Yeast	Icosahedral/spherical
Pseudoviridae	*Hemivirus*	ssRNA (+)	Protista	Icosahedral/spherical
	*Pseudovirus*	ssRNA (+)	Fungi	Icosahedral/spherical
	*Pseudovirus*	ssRNA (+)	Protista	Icosahedral/spherical
Metaviridae	*Metavirus*	ssRNA (+)	Fungi	Uncertain
	*Metavirus*	ssRNA (+)	Protista	Uncertain
Narnaviridae	*Mitovirus*	ssRNA (+)	Fungi	Unencapsidated

^1^List does not include numerous unclassified viruses.

^2^Addenda to classifications are supplied where present. These include (C.) order *Caudovirales*, (G.) subfamily *Gokushovirinae*, (P.) order *Picornavirales*, (Se.) subfamily *Sedoreovirinae*, (Sp.) subfamily *Spinareovirinae*, (T.) order *Tymovirales.*

^3^Domain Archaea, domain Bacteria, kingdom Fungi, or kingdom Protista, the latter of Whittaker's [83] Five-Kingdom System.

^4^Contains approximately 1 kb of dsDNA region within approximately 6 kb genomes; may also be listed as *Bacillariodnavirus*, in either case serving as conjunctions of “*Bacillariophyta*”, “DNA”, and “virus”.

^5^This taxon is not ICTV listed.

^6^Bacillariornaviridae *Bacillariornavirus* is listed as the taxonomic description of *Chaetoceros socialis f. radians* RNA virus, a diatom virus, by both the RNA Virus Database (http://newbioafrica.mrc.ac.za/rnavirusdb/) and NCBI. In Tomaru et al. [84] the isolation of this virus is described and they propose there its classification also into family Bacillariornaviridae. The same virus, however, is indicated as belonging to the genus *Bacillarnavirus* by ICTV, with unassigned family, and so too does Tomaru et al. [85]. The type species of this group of viruses, *Rhizosolenia setigera* RNA virus 01 (also a diatom virus), as well as *Chaetoceros tenuissimus* RNA virus 01, neither of which is indexed by NCBI, appears to be associated exclusively with *Bacillarnavirus* both by ICTV and online sources. In any case, the terms are conjunctions of “*Bacillariophyta*”, “RNA”, and “virus”.

^7^This taxon is not ICTV listed.

^8^This taxon is not ICTV listed.

**Table 2:** Representative sequenced archaeal viruses emphasizing diversity of virion structure.

Virus host^9^	Virus name	Family (genus) [description]	Type^10^	Genome	Size (GenBank accession number)	Source	Reference^11^
(C.) *Acidianus convivator *	*Acidianus* bottle-shaped virus (ABV)	Ampullaviridae (*Ampullavirus*)	Yes	dsDNA linear	23,814 nt (NC_009452)	Solfatara volcano water reservoir, Pozzuoli, Italy	Peng et al. [86]

(C.) *Acidianus convivator *	*Acidianus* two-tailed virus (ATV)	Bicaudaviridae (*Bicaudavirus*) [lemon shaped prior to growing tails]	Yes	dsDNA circular	62,730 nt (NC_007409)	Hot (87–93^°^C) acidic (pH 1.5–2) spring, solfataric field, Pozzuoli, Naples, Italy	Prangishvili et al. [87]

(C.) *Acidianus hospitalis *	*Acidianus* filamentous virus 1 (AFV1)	Lipothrixviridae ^12^ (*Gammalipothrixvirus*)	Yes	dsDNA linear	20,869 nt (NC_005830)	Acidic hot spring (85^°^C, pH 2), Crater Hills region, YNP^13^	Bettstetter et al. [88]

(C.) *Acidianus* sp.	*Acidianus* filamentous virus 2 (AFV2)	Lipothrixviridae (*Deltalipothrixvirus*)	Yes	dsDNA linear	31,787 nt (NC_009884)	Lake in crater, Solfatara volcano, Pozzuoli, Italy, with underlying hot springs (87–93^°^C, pH 1.5–2)	Häring et al. [89, 90]

(C.) *Aeropyrum pernix *	*Aeropyrum pernix* bacilliform virus 1 (APBV1)	Clavaviridae (*Clavavirus*)	Yes	dsDNA circular	5,278 nt (AB537968)	Coastal hot spring in Yamagawa, Ibusuki City, Kagoshima, Japan.	Mochizuki et al. [91]

(C.) *Pyrobaculum and Thermoproteus *	*Pyrobaculum* spherical virus (PSV)	Globuloviridae (*Globulovirus*)	Yes	dsDNA linear	28,337 nt (NC_005872)	Bioreactor based on Obsidian Pool, YNP	Häring et al. [92]

(C.) *Sulfolobus islandicus *	*Sulfolobus islandicus* filamentous virus (SIFV)	Lipothrixviridae (*Betalipothrixvirus*)	Yes	dsDNA linear	40,900 nt (NC_003214)	Solfataric fields, Iceland	Arnold et al. [93]

(C.) *Sulfolobus islandicus *	*Sulfolobus islandicus* rod-shaped virus 2 (SIRV2)	Rudiviridae (*Rudivirus*)	Yes	dsDNA linear	35,450 nt (NC_004086)	Solfataric fields, Iceland	Prangishvili et al. [94]

(C.) *Sulfolobus neozealandicus *	*Sulfolobus neozealandicus* ^14^ droplet-shaped virus (SNDV)	Guttaviridae (*Guttavirus*)	Yes	dsDNA circular	20 kb (not sequenced)	Isolated from carrier state^15^ with host	Arnold et al. [95]

(C.) *Sulfolobus shibatae* ^16^	*Sulfolobus* spindle-shaped virus 1 (SSV1)^17^	Fuselloviridae (*Fusellovirus*)	Yes	dsDNA circular	15,465 nt (NC_001338)	Lysogen isolated from Beppu Hot Springs, Japan	Schleper et al. [96]; Yeats et al. [97]

(C.) *Sulfolobus solfataricus *	*Sulfolobus* turreted icosahedral virus (STIV)	Unclassified [icosahedral, “turret”-like projections, surrounding lipid that in turn surrounds viral DNA]		dsDNA circular	17,663 nt (NC_005892)	Acidic (pH 2.9–3.9) hot spring (72–92^°^C), Rabbit Creek Thermal Area, YNP	Rice et al. [98]

(C.) *Thermoproteus tenax *	*Thermoproteus tenax* virus 1 (TTV1)	Lipothrixviridae (*Alphalipothrixvirus*)	Yes	dsDNA linear	13,669 nt (X14855)	Lysogen isolated from mud hole (93^°^C, pH 6), Krafla, Iceland	Janekovic et al. [99]

(E.) *Haloarcula hispanica *	His1 virus	[spindle/lemon-shaped, short “tail”-like fiber] (*Salterprovirus*)	Yes	dsDNA linear	14,462 nt (NC_007914)	Hypersaline water, Avalon Saltern, Corio Bay, Victoria, Australia	Bath and Dyall-Smith [100]

(E.) *Haloarcula hispanica *	Halovirus SH1 (a.k.a., *Haloarcula* phage SH1)	Unclassified [icosahedral and lipid containing]		dsDNA linear	30,889 nt (NC_007217)	Hypersaline, Serpentine Lake, Rottnest Island, Western Australia, Australia	Porter et al. [101]

(E.) *Halorubrum coriense *	*Halorubrum* phage HF2	Myoviridae [=contractile tail] (Φ*H-like viruses*)		dsDNA linear	77,670 nt (NC_003345)	Saltern, Geelong, Victoria, Australia	Tang et al. [102]

(E.) *Halorubrum* sp.	*Halorubrum* pleomorphic virus 1 (HRPV1)	Unclassified [lipid-containing virion]		ssDNA circular	7,048 nt (NC_012558)	Solar saltern, Trapani, Sicily, Italy	Pietilä et al. [103]

(E.) *Methanobacterium thermoautotrophicum *Marburg	*Methanobacterium* phage *ψ*M1	Siphoviridae [=long, non-contractile tail] (*ψ*M1*-like viruses*)	Yes	dsDNA linear	>26,111 nt^18^ (NC_001902)	Experimental autodigester (55–60^°^C)	Meile et al. [104]; Pfister et al. [105]

^9^(C.): Crenarchaeota. (E.): Euryarchaeota.

^10^Viral type species.

^11^Additional references were also consulted in assembling the table, particularly Pina et al. [39] and Krupovic et al. [40].

^12^Members of family Lipothrixviridae have enveloped, filamentous virions.

^13^Yellowstone National Park, USA.

^14^More often found as *Sulfolobus newzealandicus* but is *S. neozealandicus* in the original publication.

^15^Carrier state can refer to a number of different phenomena including chronic infections, lytic infection of only a fraction of bacteria in culture, or unstable lysogeny [106] but with archaeal viruses the meaning tends to be synonymous with chronic infections [39].

^16^Described as *S. shibatae* B12 by Schleper et al. [96] but as *S. acidocaldarius* B12 by both Martin et al. [107] and Yeats et al. [97].

^17^Originally called SAV1 for *S. acidocaldarius* virus [107].

^18^Archaeal virus *ψ*M2 is a spontaneous deletion mutant of *ψ*M1 (at least 0.7 kb missing), and we have listed *ψ*M2's genome length and accession number; within virions, *ψ*M1 possesses ~3 kb of circular redundancy, and as reviewed by Pfister et al. [105], “phage particles have been shown by electron microscopy to contain 30.4 ± 1.0 kb of DNA.”

**Table 3:** Notable viruses of protists.

Virus name	Family	Type^19^	Genome type (segments), morphology, size, GenBank accession number or reference (if available)	Host type
	Genus			
*Paramecium bursaria* Chlorella Virus 1	Phycodnaviridae *Chlorovirus *	Yes	dsDNA, icosahedral, 330,611 nt (NC_000852)	Algae
*Emiliania huxleyi * Virus 86	Phycodnaviridae *Coccolithovirus *	Yes	dsDNA, icosahedral, 407,339 nt (NC_007346)	Algae
*Micromonas pusilla * Virus SP1	Phycodnaviridae *Prasinovirus *	Yes	dsDNA, icosahedral (NCBI taxonomic ID = 373996)	Algae
*Chrysochromulina brevifilum * Virus PW1	Phycodnaviridae *Prymnesiovirus *	Yes	dsDNA, icosahedral (NCBI taxonomic ID = 352209)	Algae
*Heterosigma akashiwo * Virus 01	Phycodnaviridae *Raphidovirus *	Yes	dsDNA, icosahedral (NCBI taxonomic ID = 97195)	Algae
*Heterocapsa circularisquama* DNA Virus 01	Unassigned *Dinodnavirus *	Yes	dsDNA, complex, lipid-containing, icosahedral based (NCBI taxonomic ID = 650121)	Algae
*Chaetoceros salsugineum* DNA Virus 01	Unassigned *Bacilladnavirus* ^20^	Yes	ssDNA, icosahedral	Algae
*Micromonas pusilla* Reovirus	Reoviridae *Mimoreovirus *	Yes	dsRNA (11 segments), icosahedral, 25563 nt (NC_008177, NC_008178, NC_008171, NC_008180, NC_008179, NC_008176, NC_008181, NC_008172, NC_008173, NC_008174, NC_008175)	Algae
*Heterocapsa circularisquama *RNA Virus 01	Alvernaviridae *Dinornavirus *	Yes	ssRNA (+), icosahedral	Algae
*Aurantiochytrium* single-stranded RNA virus	Labyrnaviridae *Labyrnavirirus * ^21^		ssRNA (+), icosahedral, 9035 nt (NC_007522)	Algae
*Heterosigma akashiwo* RNA virus	Marnaviridae *Marnavirus *	Yes	ssRNA (+), icosahedral, 8,587 nt (NC_005281)	Algae
*Volvox carteri *Lueckenbuesser virus	Pseudoviridae *Hemivirus *		ssRNA (+), icosahedral/spherical	Algae
*Volvox carteri* Osser virus	Pseudoviridae *Hemivirus *		ssRNA (+), icosahedral/spherical	Algae
*Rhizosolenia setigera* RNA Virus 01	Unassigned *Bacillarnavirus* ^22^	Yes	ssRNA (+), icosahedral	Algae
*Physarum polycephalum * Tp1 virus	Pseudoviridae *Pseudovirus *		ssRNA (+), icosahedral/spherical	Cellular slime mold
*Acanthamoeba polyphaga* Mimivirus	Mimiviridae *Mimivirus *	Yes	dsDNA, complex, lipid-containing, icosahedral based, 1,181,549 nt (NC_014649)	Protozoa
*Megavirus chilensis*	Mimiviridae *Mimivirus *		dsDNA, icosahedral, 1,259,197 nt (JN258408)	Protozoa
*Acanthamoeba castellanii* mamavirus strain Hal-V	Mimiviridae (unassigned genus)		dsDNA, icosahedral, 1,191,693 nt (JF801956)	Protozoa
*Acanthamoeba polyphaga* Moumouvirus Monve isolate Mv13-mv	Mimiviridae (unassigned genus)		dsDNA, icosahedral, ~1,015,033 nt (calculated from multiple contig sequences; JN885994–JN886001)	Protozoa
*Acanthamoeba polyphaga* Megavirus courdo7 isolate Mv13-c7	Mimiviridae (unassigned genus)		dsDNA, icosahedral, ~1,170,106 nt (calculated from multiple contig sequences; JN885990–JN885993)	Protozoa
*Cafeteria roenbergensis* virus BV-PW1	Mimiviridae (unassigned genus)		dsDNA, icosahedral, 617,453 nt (NC_014637)	Protozoa
*Marseillevirus*	Marseilleviridae *Marseillevirus * ^23^	Yes^24^	dsDNA, icosahedral, 368,454 nt (NC_013756)	Protozoa
*Lausannevirus*	Marseilleviridae *Marseillevirus *		dsDNA, icosahedral, 346,754 nt (NC_015326)	Protozoa
*Giardia lamblia* virus	Totiviridae *Giardiavirus *	Yes	dsRNA, icosahedral, 6,277 nt (NC_003555)	Protozoa
*Leishmania* RNA virus 1-1	Totiviridae *Leishmaniavirus *	Yes	dsRNA, icosahedral, 5,284 nt (NC_002063)	Protozoa
*Trichomonas vaginalis * Virus 1	Totiviridae *Trichomonasvirus *	Yes	dsRNA, icosahedral, 4,657 nt (JF436869)	Protozoa
*Cryptosporidium parvum * Virus 1	Partitiviridae *Cryspovirus *	Yes	dsRNA, icosahedral [108]	Protozoa
*Phytophthora *Endornavirus 1	Endornaviridae *Endornavirus *		dsRNA, unencapsidated, 13,883 nt (AJ877914)	Water mold
*Rhizidiomyces* virus	Unassigned *Rhizidiovirus *	Yes	dsRNA, icosahedra [109]	Water mold

^19^Viral type species.

^20^Contains approximately 1 kb of dsDNA region within approximately 6 kb genomes; may also be listed as *Bacillariodnavirus*, in either case serving as conjunctions of “*Bacillariophyta*”, “DNA”, and “virus”.

^21^These taxa are not ICTV listed.

^22^See Table 1 for further discussion of this taxon.

^23^Proposed taxa; see text.

^24^Proposed type species.

**Table 4:** Notable viruses of yeasts, molds, and pathogenic fungi.

Virus name	(Order) family [subfamily]	Type^25^	Genome type (segments), morphology, size, GenBank	Host type
	Genus		accession number or reference (if available)	
*Saccharomyces cerevisiae * Virus L-A (L1)	Totiviridae *Totivirus *	Yes	dsRNA (1), icosahedral, 4,579 nt (NC_003745)	Yeast

*Saccharomyces cerevisiae * Ty5 virus	Pseudoviridae *Hemivirus *	Yes	ssRNA (+) (1), icosahedral/spherical	Yeast

*Saccharomyces cerevisiae * Ty1 virus	Pseudoviridae *Pseudovirus *	Yes	ssRNA (+) (1), icosahedral/spherical	Yeast

*Saccharomyces cerevisiae * Ty3 virus	Metaviridae *Metavirus *	Yes	ssRNA (+) (1), uncertain	Yeast

*Saccharomyces* 20S RNA narnavirus	Narnaviridae *Narnavirus *	Yes	ssRNA (+) (1), unencapsidated, 2,514 nt (NC_004051)	Yeast

*Aspergillus ochrace us* virus	Partitiviridae *Partitivirus *		dsRNA (1), icosahedral	Mold^26^

*Penicillium chrysogenum* virus	Chrysoviridae *Chrysovirus *	Yes	dsRNA (4), icosahedral, 12,640 nt (NC_007542, NC_007539, NC_007541, NC_007540)	Mold

*Fusarium solani * Virus 1 (a.k.a., mycovirus FusoV)	Partitiviridae *Partitivirus *		dsRNA (2), icosahedral, 3,090 nt (NC_003886, NC_003885)	Human pathogen

*Tolypocladium cylindrosporum * virus 1	Totiviridae *Totivirus *		dsRNA (1), icosahedral, 5,196 nt (NC_014823)	Mosquito pathogen

*Helminthosporium victoriae * Virus 190S	Totiviridae *Victorivirus *	Yes	dsRNA (1), icosahedral, 5,179 nt (NC_003607)	Plant pathogen

*Cryphonectria* hypovirus 1	Hypoviridae *Hypovirus *	Yes	dsRNA (1), pleomorphic cytoplasmic vesicles, 12,734 nt (NC_001492)	Plant pathogen

*Helicobasidium mompa* endornavirus 1	Endornaviridae *Endornavirus *		dsRNA (1), unencapsidated, 16,614 nt (NC_013447)	Plant pathogen

Mycoreovirus 1 (of *Cryphonectria parasitica*)	Reoviridae [Spinareovirinae] *Mycoreovirus *	Yes	dsRNA (11), spherical, double shelled, 23,433 nt (NC_010743, NC_010744, NC_010745)	Plant pathogen

*Rosellinia necatrix * Megabirnavirus 1	Megabirnaviridae *Megabirnavirus *	Yes	dsRNA (2), spherical^27^, 16,111 nt [110]	Plant pathogen

*Rosellinia necatrix * Quadrivirus 1	Unassigned		dsRNA (4), spherical, 17,078 nt (NC_016757, NC_016759, NC_016760, NC_016758) [111]	Plant pathogen

*Sclerotinia sclerotiorum * Hypovirulence-associated DNA virus 1	Unassigned [Geminiviridae-like]		ssDNA (1), spherical or icosahedral, 2166 nt [112]	Plant pathogen

*Sclerotinia sclerotiorum * Debilitation-associated RNA virus	(Tymovirales) Alphaflexiviridae *Sclerodarnavirus *	Yes	ssRNA (+) (1), filamentous, 5,470 nt (NC_007415)	Plant pathogen

*Botrytis * Virus F	(Tymovirales) Gammaflexiviridae *Mycoflexivirus *	Yes	ssRNA (+) (1), filamentous, 6,827 nt (NC_002604)	Plant pathogen

*Botrytis * Virus X	(Tymovirales) Alphaflexiviridae *Botrexvirus *	Yes	ssRNA (+) (1), filamentous, 6,966 nt (NC_005132)	Plant pathogen

*Cryphonectria* Mitovirus 1	Narnaviridae *Mitovirus *	Yes	ssRNA (+) (1), unencapsidated	Plant pathogen

^25^Viral type species.

^26^Host is *Aspergillus ochraceus*.

^27^Chiba et al. [110, 111] describe the virions as “spherical” but the published electron micrographs are also suggestive of icosahedral.
